# Mutation in *Prkra* results in cerebellar abnormality and reduced eIF2α phosphorylation in a model of DYT-PRKRA

**DOI:** 10.1242/dmm.050929

**Published:** 2024-11-26

**Authors:** Samuel B. Burnett, Allison M. Culver, Tricia A. Simon, Taylor Rowson, Kenneth Frederick, Kristina Palmer, Stephen A. Murray, Shannon W. Davis, Rekha C. Patel

**Affiliations:** ^1^ Department of Biological Sciences, University of South Carolina, Columbia, SC 29208, USA; ^2^ Genetic Resource Center, The Jackson Laboratory, 600 Main Street, Bar Harbor, ME 04609, USA

**Keywords:** PACT/RAX, PKR, EIF2AK2, Dystonia, DYT-PRKRA, Cerebellum

## Abstract

Variants in the *PRKRA* gene*,* which encodes PACT, cause the early-onset primary dystonia DYT-PRKRA, a movement disorder associated with disruption of coordinated muscle movements. PACT and its murine homolog RAX activate protein kinase R (PKR; also known as EIF2AK2) by a direct interaction in response to cellular stressors to mediate phosphorylation of the α subunit of eukaryotic translation initiation factor 2 (eIF2α). Mice homozygous for a naturally arisen, recessively inherited frameshift mutation, *Prkra^lear-5J^*, exhibit progressive dystonia. In the present study, we investigated the biochemical and developmental consequences of the *Prkra^lear-5J^* mutation. Our results indicated that the truncated PACT/RAX protein retains its ability to interact with PKR but inhibits PKR activation. Mice homozygous for the mutation showed abnormalities in cerebellar development as well as a severe lack of dendritic arborization of Purkinje neurons. Additionally, reduced eIF2α phosphorylation was noted in the cerebellum and Purkinje neurons of the homozygous *Prkra^lear-5J^* mice. These findings indicate that PACT/RAX-mediated regulation of PKR activity and eIF2α phosphorylation plays a role in cerebellar development and contributes to the dystonia phenotype resulting from the *Prkra^lear-5J^* mutation.

## Teaser

**Summary:** This study shows, for the first time, a role of reduced eIF2α phosphorylation in the early-onset primary dystonia DYT-PRKRA and in cerebellar development in a mouse model.

## INTRODUCTION

Dystonia is a movement disorder involving sustained muscle contractions, which can lead to painful, twisting, repetitive movements and abnormal postures (Geyer and Bressman, 2006; Bragg et al., 2011). There are multiple underlying etiologies; although several dystonia-causing variants have been identified in various genes, the underlying pathological molecular mechanisms for most dystonia types remain unknown (Thomsen et al., 2024). The advent of next-generation sequencing technologies has made it efficient to identify the genes associated with dystonia, and this list is currently growing steadily (Thomsen et al., 2024). Characterizing the genetic causes of inherited forms of dystonia offers the opportunity to investigate the underlying molecular pathomechanisms. The focus of our research presented here is DYT-PRKRA [also known as dystonia 16 (DYT16)], which is an early-onset, generalized dystonia caused by variants in the *PRKRA* gene, which encodes the protein PACT (Patel and Sen, 1998), a stress-modulated activator of the protein kinase PKR (Patel et al., 2000). The murine homolog of PACT is termed RAX (Ito et al., 1999), and because most of the work on PACT so far has been in human cells, we will refer to *Prkra*-encoded murine protein as PACT/RAX.

PKR is a ubiquitously expressed double-stranded RNA (dsRNA)-activated protein kinase (Meurs et al., 1990; Garcia et al., 2007), active under cellular stress conditions such as viral infections, oxidative and endoplasmic reticulum (ER) stress, and serum or growth factor deprivation (Ito et al., 1999; Patel et al., 2000). In virally infected cells, PKR is activated by direct interactions with dsRNA, a viral replication intermediate for many viruses (Barber, 2001), but, in the absence of viral infections, other stress signals activate PKR via its protein activator PACT/RAX (Patel and Sen, 1998) in a dsRNA-independent manner. Two evolutionarily conserved dsRNA-binding motifs (dsRBM1 and dsRBM2) in PKR allow for its interactions with dsRNA (Feng et al., 1992; Green and Mathews, 1992; Patel and Sen, 1992) as well as its dsRNA-independent interaction with PACT/RAX (Peters et al., 2001; Huang et al., 2002) and other regulatory proteins (Chang and Ramos, 2005). Upon binding dsRNA or PACT/RAX, PKR is activated via a conformational change and autophosphorylation (Nanduri et al., 1998; Cole, 2007). In the absence of stress, PKR stays inactive via direct interactions with the transactivation response element (TAR) RNA-binding protein (TRBP; also known as TARBP2) mediated by the dsRBMs of each protein (Benkirane et al., 1997; Laraki et al., 2008). TRBP inhibits PKR via the formation of both TRBP-PACT/RAX and TRBP-PKR heterodimers (Daher et al., 2009; Singh et al., 2011; Singh and Patel, 2012). Our previous work has established that the regulation of PKR activation in response to stress depends on shifting the PKR inhibitory (TRBP-PACT/RAX and TRBP-PKR) interactions to PKR-activating (PACT/RAX-PKR and PACT/RAX-PACT/RAX) interactions in response to the stress signal (Daher et al., 2009). This is regulated by stress-induced PACT/RAX phosphorylation, which dissociates PACT/RAX from TRBP and allows for its interaction with PKR (Singh et al., 2011; Singh and Patel, 2012).

PKR is one of the four protein kinases that regulate the integrated stress response (ISR), an evolutionarily conserved pathway activated by a diverse set of stress signals in eukaryotic cells. ISR works to restore the cellular homeostasis (Pakos-Zebrucka et al., 2016) by reducing the rate of general protein synthesis while allowing selective synthesis of proteins involved in cellular recovery. The central regulatory step in this pathway is the phosphorylation of the α subunit of eukaryotic translation initiation factor 2 (eIF2α) on serine 51 by one of the four serine/threonine kinases (Donnelly et al., 2013; Taniuchi et al., 2016). Phosphorylation of eIF2α prevents the formation of the ternary complex required for translation initiation, leading to a decrease in overall protein synthesis while allowing translation of selected mRNAs that have short upstream open-reading frames (ORFs) in their 5′ untranslated region (UTR) and encode proteins that aid in cellular recovery (Wek, 2018). Although transient eIF2α phosphorylation promotes cellular survival, prolonged eIF2α phosphorylation is pro-apoptotic owing to transcriptional upregulation as well as preferential translation of pro-apoptotic proteins (Donnelly et al., 2013). Thus, although ISR is primarily a response to restore cellular homeostasis and promote survival, exposure to severe or prolonged chronic stress drives signaling towards cellular death.

DYT-PRKRA is an early-onset movement disorder characterized by progressive limb dystonia, laryngeal and oromandibular dystonia and parkinsonism (Camargos et al., 2008). Ten PACT variants leading to DYT-PRKRA have been reported [Online Mendelian Inheritance in Man (OMIM): dystonia 16 (DYT16), #612067] worldwide (Camargos et al., 2008, 2012; Seibler et al., 2008; Lemmon et al., 2013; Zech et al., 2014; de Carvalho Aguiar et al., 2015; Quadri et al., 2016; Dos Santos et al., 2018; Masnada et al., 2021; Bhowmick et al., 2022). Using DYT-PRKRA patient-derived lymphoblasts and other *in vitro* biochemical approaches, our laboratory has established that PACT variants increase cellular susceptibility to ER stress due to dysregulated eIF2α stress response signaling (Vaughn et al., 2015; Burnett et al., 2019; Burnett et al., 2020). In agreement with our findings, the dysregulation of eIF2α signaling was also reported in DYT-TOR1A, DYT-THAP1, DYT-SGCE and sporadic cervical dystonia (Rittiner et al., 2016; Beauvais et al., 2018; Zakirova et al., 2018). Collectively, these findings indicate a potential common pathological link among some forms of inherited dystonia.

Palmer et al. described a spontaneous frameshift mutation (*lear-5J*) in the murine *Prkra* gene that codes for RAX, the mouse homolog of PACT (Ito et al., 1999; Palmer et al., 2016). This frameshift mutation results in truncation of the PACT/RAX protein within the dsRBM2 domain (Huang et al., 2002). PACT and RAX are highly homologous, differing only in six amino acids, four of which are conservative changes (Patel and Sen, 1998; Ito et al., 1999). The *Prkra^lear-5J^* mice present with craniofacial developmental abnormalities, small ear size (giving the name ‘lear’ for ‘little ears’), drastically reduced body size, kinked tails, and ascending dystonia that progresses until becoming fatal at 3-6 weeks of age (Palmer et al., 2016). In the present study, we undertook an initial characterization of the *in vitro* and *in vivo* consequences of the *Prkra^lear-5J^* mutation. Our results demonstrated that the truncated protein is present in mouse brain but not in murine embryonic fibroblasts (MEFs), and that the mutant PACT/RAX protein retains its ability to interact with PKR and inhibits PKR activation. Our *in vivo* data evaluating the brains of *Prkra^lear-5J^* mice demonstrate defective foliation of the cerebellum and a dramatic reduction in dendritic arborization of Purkinje neurons. Finally, we also observed reduction in phosphorylated eIF2α and elevated expression of CreP (also known as *Ppp1r15b*), an eIF2α-specific phosphatase. These results demonstrate that dysregulating PACT/RAX-mediated eIF2α phosphorylation in mouse has severe consequences on cerebellar development and could contribute to the etiology of the observed dystonia phenotype.

## RESULTS

### A single-nucleotide insertion truncates PACT/RAX in dsRBM2 in *Prkra^lear-5J^* mice

**Fig. 1.**
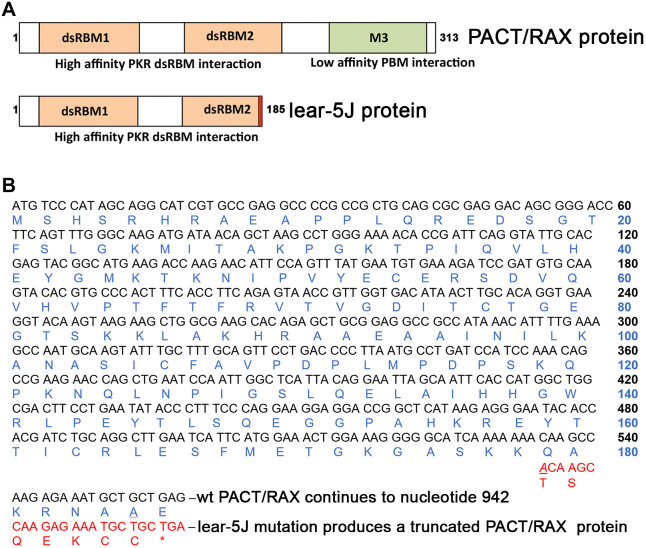
**Schematic representations of the lear-5J frameshift mutation in the *Prkra* gene.** (A) Functional domains of PACT/RAX and lear-5J truncated protein. Orange boxes, conserved dsRBM1 and dsRBM2, which facilitate high-affinity double-stranded RNA (dsRNA) as well as protein-protein interactions; green box, dsRBM3, which does not bind dsRNA but has weak binding affinity to the PBM within the catalytic (kinase) domain of PKR. The frameshift mutation from a single-nucleotide insertion results in the addition of seven novel amino acids represented in red before the stop codon. dsRBM, dsRNA-binding motif; PBM, PACT/RAX-binding motif. (B) Frameshift mutation in *Prkra* open-reading frame. A single adenine insertion at nucleotide position 534 (underlined) results in a truncated protein with the original 178 amino acids of PACT/RAX followed by seven novel amino acids before a premature stop codon (asterisk) truncating the protein. This truncation occurs within the dsRBM2 functional domain.

Palmer et al. identified a recessively inherited spontaneous mutation on the BTBR *T*^+^ *Itpr3^tf^*/J genetic background, which resulted in small body size, small ears, kinked tails, progressive dystonia or paralysis, hearing loss and mortality (Palmer et al., 2016). They confirmed by sequence analysis that these phenotypes arose from a single adenine insertion in codon 178 of the *Prkra* gene. The frameshift caused by the insertion leads to a premature stop codon after seven extraneous amino acids within the dsRBM2, one of the functional motifs of PACT/RAX (Fig. 1A,B). The resultant protein contains the entire dsRBM1 and a partial dsRBM2 eliminating some of the residues within this domain known to be important for dsRNA-binding and protein-protein interactions. However, the truncated protein retains the entire dsRBM1, which is most crucial for dsRNA-binding and protein-protein interactions based on the previous work from our group and other laboratories (Peters et al., 2001; Huang et al., 2002; Chukwurah et al., 2018).

### Effects of the *Prkra^lear-5J^* mutation on PACT/RAX dsRNA binding and PKR interaction

**Fig. 2.**
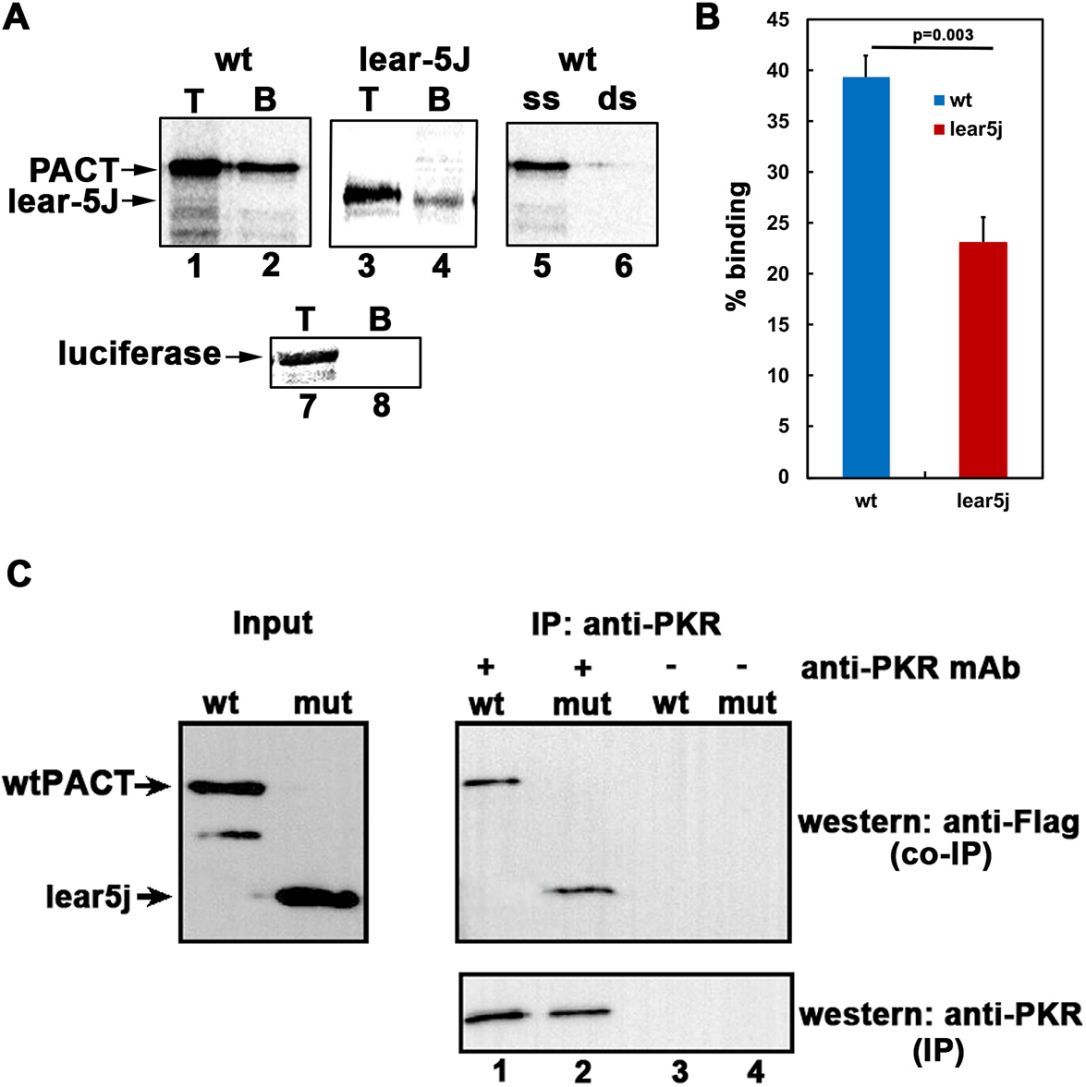
**The lear-5J protein binds dsRNA less efficiently but interacts with PKR similarly to wt PACT/RAX.** (A) dsRNA-binding activity of wt PACT/RAX and lear-5J truncated protein was measured by a poly(I):poly(C)-agarose-binding assay with *in vitro* translated ^35^S-labeled proteins. T, total input; B, proteins bound to poly(I):poly(C)-agarose. Competition lanes 5 and 6: competition with 100-fold molar excess of single-stranded RNA (ss) or dsRNA (ds). The faint band below the parent PACT/RAX band represents products of *in vitro* translation from an internal methionine codon in lane 1. (B) Quantification of the dsRNA-binding assay. Bands were quantified by phosphorimaging analysis, and percentage bound was calculated. Error bars: s.d. from three independent experiments. The *P*-value (0.003) calculated using statistical analyses indicated significant difference between percentage dsRNA-binding of wt (blue bar) and that of lear-5J mutant (red bar). (C) Co-immunoprecipitation of endogenous PKR and Flag-PACT/RAX or Flag-lear-5J overexpressed in HeLa cells. HeLa cells were transfected with Flag wt PACT/RAX or Flag-lear-5J in pCDNA3.1 expression constructs at 40% confluency and harvested 24 h post-transfection. Whole-cell extracts were immunoprecipitated at 4°C overnight using 100 ng anti-PKR antibody per immunoprecipitation. Samples were then analyzed via SDS-PAGE gel electrophoresis and western blot analysis probing for Flag-tagged wt PACT/RAX or lear-5J (co-IP panel) using monoclonal anti-Flag-M2 (Sigma-Aldrich) antibody. To ascertain that an equal amount of protein was immunoprecipitated, blots were re-probed using an anti-PKR antibody (IP panel). Input blots without immunoprecipitation demonstrate that equal amounts of each protein were present prior to immunoprecipitation. IP, immunoprecipitation; mAb, monoclonal antibody; mut, mutant; wt, wild type.

To characterize how the lear-5J mutation affects the properties of the protein, we tested whether the mutation affects the ability of PACT/RAX to bind dsRNA. An *in vitro* dsRNA-binding assay previously well established for PKR and PACT (Patel and Sen, 1992, 1998; Patel et al., 1996; Huang et al., 2002; Chukwurah et al., 2018) was performed using dsRNA immobilized on agarose beads and *in vitro* translated ^35^S methionine-labeled proteins. As seen in Fig. 2A and B, the *Prkra^lear-5J^* mutant protein showed reduced dsRNA binding in comparison to the wild-type (wt) PACT/RAX (Fig. 2A, lanes 2 and 4, Fig. 2B). Approximately 40% of the wt PACT/RAX protein bound to the dsRNA-agarose beads, but only ∼23% of *Prkra^lear-5J^* protein showed binding. To ascertain the specificity of the dsRNA-binding assay, we used *in vitro* translated firefly luciferase as a negative control, which does not bind to dsRNA (Fig. 2A, lanes 7 and 8). Additionally, we demonstrated the specificity of the interaction for dsRNA by adding excess dsRNA or single-stranded RNA (ssRNA) as competitors. As seen in lanes 5 and 6 in Fig. 2A, the binding to dsRNA immobilized on beads could be effectively competed by exogenously added dsRNA but not ssRNA. This ascertained the specificity of the dsRNA-binding assay, and it could be concluded that the *Prkra^lear-5J^*-derived mutant protein showed binding to dsRNA at a reduced efficiency compared to that of the wt PACT/RAX protein.

Previously, our laboratory reported that mutating specific hydrophobic residues within the dsRBM1 of PACT significantly disrupts PACT-PKR interactions, whereas disrupting hydrophobic residues in dsRBM2 has minimal consequences for PACT-PKR interaction (Chukwurah et al., 2018). As the *Prkra^lear-5J^* mutation truncates the protein within the dsRBM2 of PACT/RAX, we tested whether the truncated protein retains its ability to interact with PKR (Fig. 2C). We performed co-immunoprecipitation (co-IP) assays on cells overexpressing either Flag-tagged wt PACT/RAX or lear-5J proteins. Our results indicated that, despite the truncation, the lear-5J protein interacted with PKR with equal binding affinity to that of wt PACT/RAX (Fig. 2C, lanes 1 and 2). In the absence of PKR antibody, we did not detect Flag-wt PACT/RAX or Flag-lear-5J, thus indicating that there is no binding to the beads nonspecifically in the absence of PKR protein (Fig. 2C, lanes 3 and 4). Lanes 1 and 2 of Fig. 2C (lower panel) indicate that an equal amount of endogenous PKR was immunoprecipitated in both samples, while lanes 3 and 4 of Fig. 2C (lower panel) indicate that PKR is absent in the absence of anti-PKR antibody. The input blot in Fig. 2C (left) demonstrates equal expression of Flag-wt PACT/RAX and Flag-lear-5J prior to immunoprecipitation. Thus, the truncated lear-5J protein retains its ability to interact with PKR.

### Truncated lear-5J protein inhibits PKR kinase activity

**Fig. 3.**
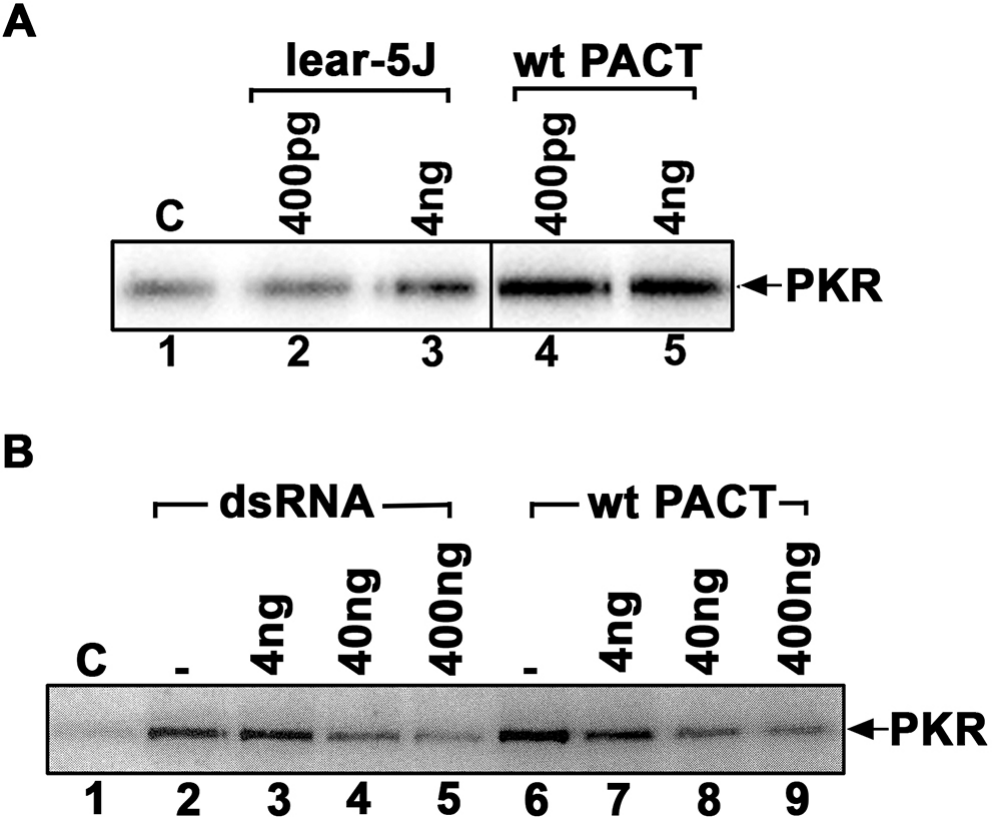
**(A) Effects of lear-5J protein on PKR kinase activity.** (A) PKR kinase activity assay was performed using PKR immunoprecipitated from HeLa cells, recombinant lear-5J and wt PACT/RAX proteins, and 1 μCi [γ-^32^P] ATP per reaction. Either pure recombinant lear-5J (left) or wt PACT/RAX (right) protein was added as activator in the amounts indicated above each lane. Phosphorylated PKR was visualized after SDS-PAGE and phosphorimager analysis. (B) The truncated lear-5J protein inhibits PKR activation. PKR immunoprecipitated from HeLa cell extracts was activated with polyI:polyC (lanes 2-5) or 4 ng recombinant pure wt PACT/RAX protein (lanes 6-9). Increasing amounts of recombinant pure lear-5J protein (4-400 ng) were added (lanes 2-9) as indicated to assess the effect on PKR activity. Lane 1, PKR activity without any added activator; lane 2, PKR activity in the presence of polyI:polyC; lanes 3-5, PKR activity in the presence of polyI:polyC and 4 ng, 40 ng or 400 ng lear-5J protein; lane 6, PKR activity in the presence of 4 ng wt PACT/RAX protein; lanes 7-9, PKR activity in the presence of 4 ng wt PACT/RAX and 4 ng, 40 ng or 400 ng lear-5J protein. Phosphorylated proteins were analyzed by SDS-PAGE and phosphorimager analysis. C, control.

We previously established that PACT/RAX activates PKR under conditions of cellular stress (Patel et al., 2000; Daher et al., 2009; Singh et al., 2009, 2011; Singh and Patel, 2012) but recombinant purified PACT/RAX protein can activate PKR robustly *in vitro* (Patel and Sen, 1998; Huang et al., 2002). Therefore, we next tested the effect of the *Prkra^lear-5J^* frameshift mutation on PKR activation by performing PKR activity assays using either purified recombinant hexahistidine-tagged wt PACT/RAX or lear-5J proteins to activate PKR. Both hexahistidine-tagged wt PACT/RAX and lear-5J proteins were expressed in bacteria and purified using affinity chromatography on Ni-agarose. Based on our previous work (Patel and Sen, 1998; Huang et al., 2002), two different amounts of purified recombinant proteins were tested for activation of PKR immunoprecipitated from HeLa cells (Fig. 3A). Our results indicated that, at lower concentration (400 pg), lear-5J protein did not activate PKR above background (Fig. 3A, lanes 1 and 2), whereas wt PACT/RAX caused robust activation of PKR (Fig. 3A, lane 4). Interestingly, lear-5J activated PKR very slightly at the higher concentration (4 ng) (Fig. 3A, lane 3). Because lear-5J had drastically reduced ability to activate PKR (Fig. 3A, lane 3) compared to wt PACT/RAX (Fig. 3A, lanes 4 and 5), we next tested whether lear-5J protein inhibited PKR activation brought about by dsRNA or PACT/RAX. We observed that lear-5J protein inhibited PKR activation brought about by dsRNA (Fig. 3B, lanes 2-5) and by PACT/RAX (Fig. 3B, lanes 6-9). These results indicated that lear-5J protein inhibits PKR activation in a dose-dependent manner. Although PACT/RAX interacts with PKR via the dsRBM1 and dsRBM2 motifs, the carboxy terminal motif M3 is required for activation of PKR kinase activity (Peters et al., 2001; Huang et al., 2002), and, as the truncated lear-5J protein lacks this motif, these results confirm the previously reported essential function of motif M3 for interacting with the PACT-binding motif (PBM) and activating PKR catalytically (Peters et al., 2009).

### MEFs isolated from *Prkra^lear-5J^* mice are resistant to ER stress-induced apoptosis

**Fig. 4.**
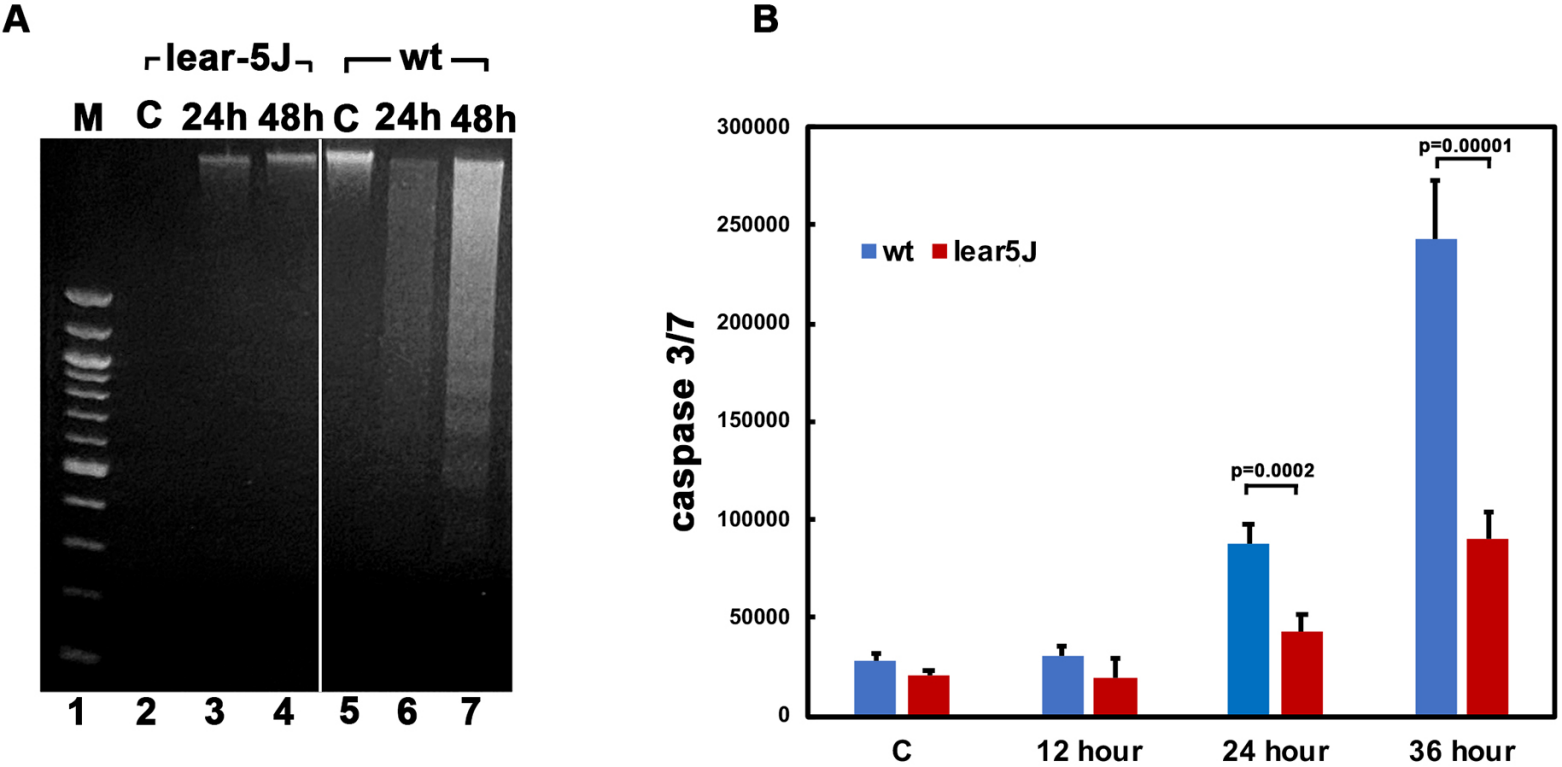
**Tunicamycin-induced apoptosis is reduced in *Prkra^lear-5J^* MEFs.** (A) DNA fragmentation analysis. Mouse embryonic fibroblasts (MEFs) established from wt (BTBR *T*^+^ *Itpr^tf^*/J) and *Prkra^lear-5J^* mice were treated with 0.5 µg/ml tunicamycin for the indicated times. Lane 1, 100 bp marker ladder (M); lanes 2-4, wt (BTBR *T*^+^ *Itpr^tf^*/J) MEFs; lanes 5-7, *Prkra^lear-5J^* MEFs. Lanes 2 and 5, untreated cells; lanes 3, 4, 6 and 7, tunicamycin-treated cells. (B) Caspase-Glo 3/7 assay. MEFs established from WT (BTBR *T*^+^ *Itpr^tf^*/J) mice and *Prkra^lear-5J^* mice were treated with 0.5 µg/ml tunicamycin for the indicated times, and caspase 3/7 activities were measured. Blue bars, wt (BTBR *T*^+^ *Itpr^tf^*/J) MEFs; red bars, *Prkra^lear-5J^* MEFs. The *P*-values that were significant are indicated.

We have previously established that PACT/RAX-induced PKR activation is involved in regulating cell survival in response to ER stress (Singh et al., 2009; Vaughn et al., 2015; Burnett et al., 2020). Thus, we compared the response of *Prkra^lear-5J^* MEFs to ER stress with that of wt (BTBR *T*^+^ *Itpr^tf^*/J) MEFs. To compare the relative apoptosis in wt and lear-5J MEFs, we used DNA fragmentation analysis in response to tunicamycin treatment. Tunicamycin treatment results in accumulation of misfolded proteins in the ER owing to inhibition of protein glycosylation and triggers ER stress response. DNA fragmentation is a late marker of apoptotic cells as the DNA is cleaved by caspase-activated DNases into nucleosomal fragments of 180 bp (Nagata et al., 2003). The wt (BTBR *T*^+^ *Itpr^tf^*/J) MEFs showed significantly high levels of DNA fragmentation in response to tunicamycin (Fig. 4A, lanes 5-7). In comparison, the *Prkra^lear-5J^* MEFs had no detectable DNA fragmentation after exposure to tunicamycin (Fig. 4A, lanes 2-4). These results indicated that *Prkra^lear-5J^* MEFs are significantly protected from ER stress-induced apoptosis.

To further validate these results, we performed caspase 3/7 activity assays under the same treatment conditions to measure apoptosis. In wt MEFs, we detected caspase activity above control levels at 24 h, which increased at 36 h post-treatment (Fig. 4B, blue bars). In contrast, the *Prkra^lear-5J^* MEFs demonstrated significantly reduced caspase activity at any of the time points post-treatment (Fig. 4B, red bars). This substantiates that the *Prkra^lear-5J^* MEFs are significantly resistant to ER stress and exhibit less apoptosis than do wt MEFs.

### Lear-5J protein is detectable in mouse brain but not in MEFs

**Fig. 5.**
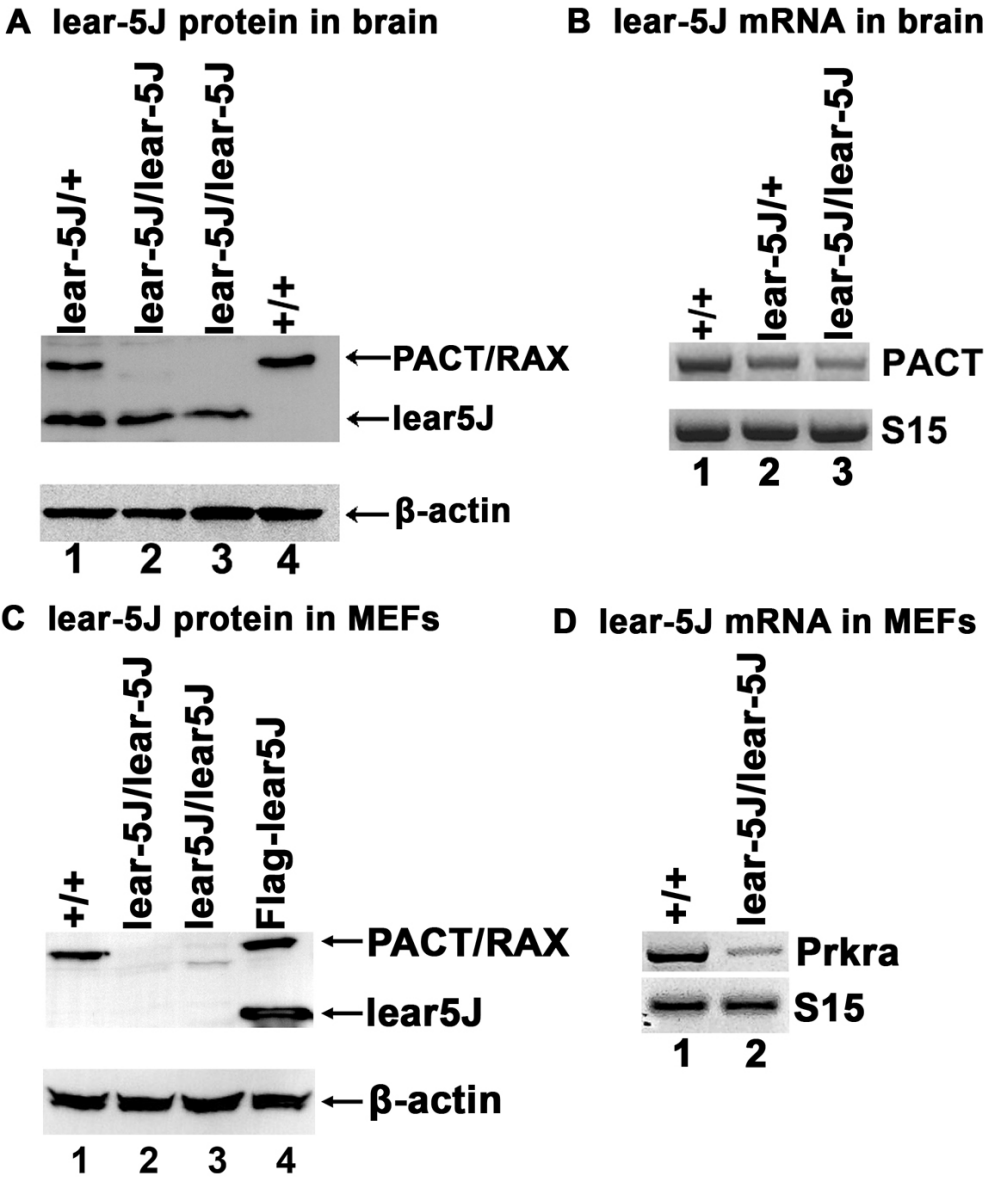
**Lear-5J truncated mutant protein is present in mouse brains but not in MEFs.** (A) Western blot analysis using brain extracts prepared from wt and *Prkra^lear-5J^* brain samples. Blots were probed for PACT/RAX using a polyclonal antibody; the best of three representative blots is shown. The position of the full-length PACT/RAX and the truncated lear-5J protein is indicated by arrows. The blots were probed with β-actin antibody, which indicates equal loading. (B) Reverse transcriptase PCR (RT-PCR) using total RNA from the brains of the indicated *Prkra^lear-5J^* genotypes using ribosomal protein S15 as the positive control to ascertain presence of an equal amount of total RNA in all samples. (C) Western blot analysis of cell extracts derived from MEFs of the indicated *Prkra^lear-5J^* genotypes and HeLa cells overexpressing Flag-lear-5J protein. Blots were probed for PACT/RAX using a polyclonal antibody; the best of three representative blots is shown. The position of the full-length PACT/RAX and the truncated lear-5J protein is indicated by arrows. (D) RT-PCR using total RNA isolated from wt and homozygous *Prkra^lear-5J^* MEFs. Ribosomal protein S15 was used as a positive control to ascertain that an equal amount of total RNA was analyzed in each sample.

The mRNAs with frameshift mutations containing an early stop codon are often degraded via nonsense-mediated decay (NMD) (Carrard and Lejeune, 2023; Patro et al., 2023; Petrić Howe and Patani, 2023). We next wanted to address whether the lear-5J mutant protein and mRNA is detectable in mice, or whether the expression of lear-5J is silenced partially or fully via NMD. To address this question, we isolated protein and total RNA from the brains of wt (BTBR *T*^+^ *Itpr^tf^*/J), heterozygous *Prkra^lear-5J^* and homozygous *Prkra^lear-5J^* mice. We performed western blot analysis utilizing a polyclonal antibody against PACT/RAX to detect the truncated lear-5J protein (Fig. 5A). wt PACT/RAX has a molecular mass of 34 kDa (Fig. 5A, upper band, lanes 1 and 4), whereas the lear-5J truncated protein has a predicted molecular mass of 22 kDa (Fig. 5A, lower band, lanes 2 and 3). Lane 1 in Fig. 5A shows that, in extract derived from *Prkra^lear-5J^* heterozygous mice, both wt PACT/RAX (upper band) and lear-5J protein (lower band) are detectable. As expected, extracts prepared from two independent *Prkra^lear-5J^* homozygous mouse brains had no detectable wt PACT/RAX band at 34 kDa; however, both had a detectable band at 22 kDa (Fig. 5A, lanes 2 and 3). Finally, we detected the presence of wt PACT/RAX, but not the lear-5J truncated protein, in extract derived from brains of wt (BTBR *T*^+^ *Itpr^tf^*/J) mice (Fig. 5A, lane 4). Blots were then probed for β-actin to ensure equal protein loading (Fig. 5A, lower panel, lanes 1-4). To further validate that the bands observed in Fig. 5A are specific to lear-5J and that the mRNA corresponding to the *Prkra* gene is present in the brain (and not degraded via NMD), we performed reverse transcriptase PCR (RT-PCR) on total RNA isolated from the brains of these mice (Fig. 5B). Our results indicated that, compared to in wt mice (Fig. 5B, lane 1), *Prkra* transcripts were present at reduced levels in *Prkra^lear-5J^* mice (Fig. 5V, lanes 2 and 3), with the greatest reduction observed in *Prkra^lear-5J^* homozygous mice (Fig. 5B, lane 3). These results indicate that although NMD may be operative on lear-5J mRNA, it does not eliminate the transcripts from the *Prkra* gene in *Prkra^lear-5J^* brains. They also confirm that the protein bands observed at the expected position in *Prkra^lear-5J^* brain extracts (Fig. 5A) are the truncated mutant lear-5J protein. RT-PCR for the ribosomal protein S15 was used as a positive control to ensure that an equal amount of cDNA was added to each PCR reaction (Fig. 5B, lower panel).

We next evaluated whether the truncated lear-5J protein was also present in *Prkra^lear-5J^* MEFs. Therefore, we performed western blot and RT-PCR analysis on MEFs derived from wt (BTBR *T*^+^ *Itpr^tf^*/J) and lear-5J mice (Fig. 5C,D). HeLa cells overexpressing Flag-lear-5J protein were used as a positive control and as a size marker for the lear-5J protein. Our results indicated that wt PACT/RAX was abundant in wt MEFs (Fig. 5C, lane 1); however, no lear-5J mutant protein was detectable in *Prkra^lear-5J^* MEFs (Fig. 5C, lanes 2 and 3). To ensure equal protein loading, we then probed for β-actin as our loading control (Fig. 5C, lower panel, lanes 1-4). Finally, we wanted to determine whether the observed absence of lear-5J protein in MEFs is due to the absence of *Prkra* mRNA, as it may be degraded completely via NMD in MEFs. We assessed this using RT-PCR as described above. Our results showed a dramatic reduction in detectable *Prkra* mRNA in MEFs homozygous for the *Prkra^lear-5J^* mutation (Fig. 5D, lane 2) compared to the wt (BTBR *T*^+^ *Itpr^tf^*/J) control (Fig. 5D, lane 1). Ribosomal protein S15 was used as a positive control to ensure that equal quantities of cDNA were used for each reaction. These results indicate that NMD may be more efficient in MEFs than in brain. In addition, these results indicate that the truncated mutant lear-5J protein may have reduced stability in MEFs as no lear-5J protein was detected in *Prkra^lear-5J^* MEFs.

### PACT/RAX is expressed in mouse cerebellum

**Fig. 6.**
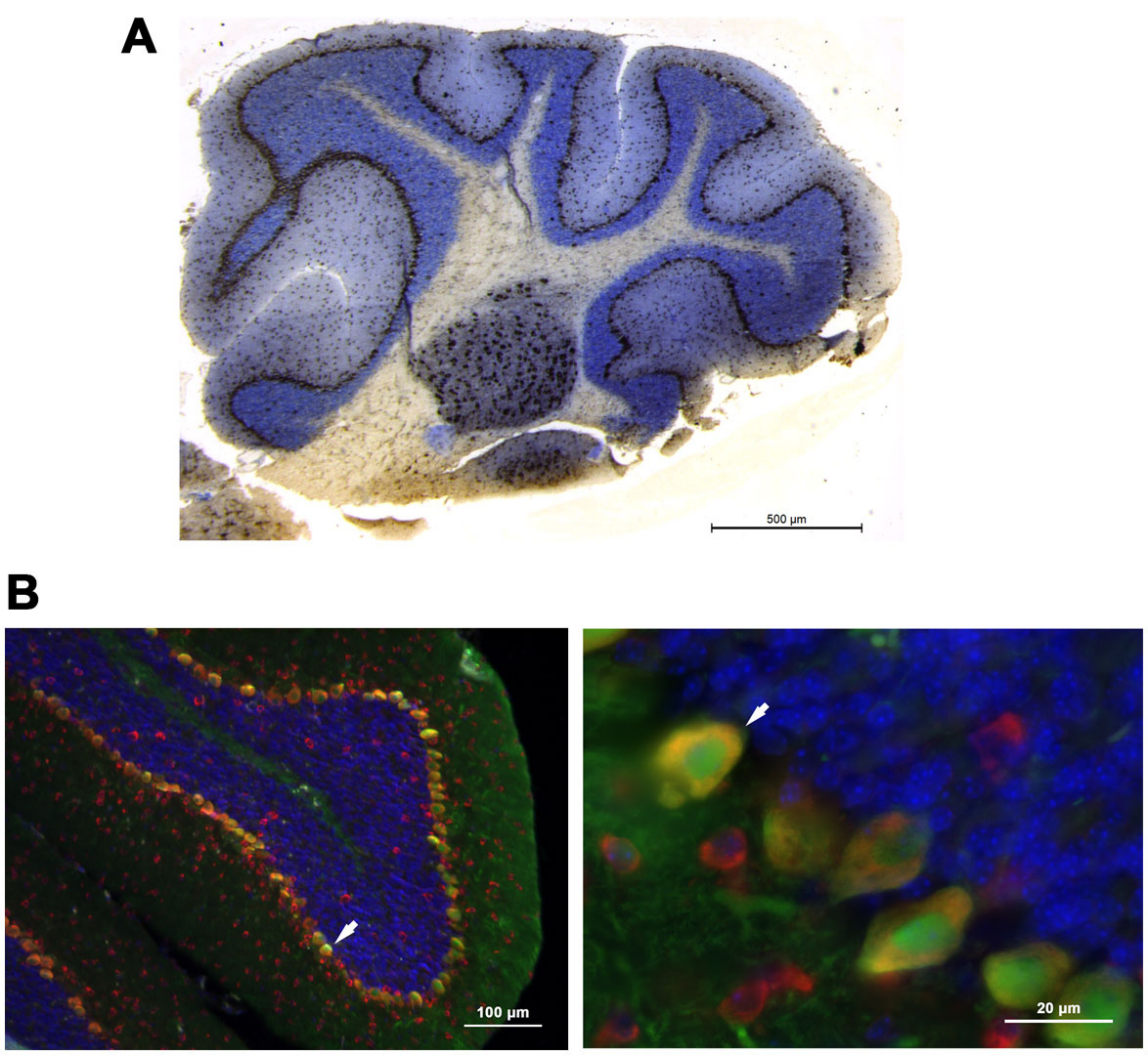
**PACT/RAX protein is abundantly expressed in mouse cerebellum and especially in Purkinje neurons.** (A) Immunohistochemistry on day 28 sagittal section of wt C57BL/6 cerebellum using anti-PACT/RAX antibody. Brown staining indicates the presence of PACT/RAX protein. (B) Immunohistochemistry of the tissue described in A, showing colocalization of PACT/RAX with a Purkinje neuron-specific marker, calbindin. Red, PACT/RAX; blue, DAPI; green, calbindin.

Previous studies have demonstrated that PACT/RAX contributes to craniofacial development (Dickerman et al., 2011; Palmer et al., 2016) and to migration of cerebellar granule neurons (Yong et al., 2015). As the *Prkra^lear-5J^* mouse exhibits dystonia, we next evaluated the presence of PACT/RAX in the cerebellum of developing wt (BTBR *T*^+^ *Itpr^tf^*/J) and *Prkra^lear-5J^* mice. The cerebellum is known for motor coordination and proprioception, and a growing number of studies have indicated a pathophysiological role of the cerebellum in dystonia (Bologna and Berardelli, 2018; Jinnah and Hess, 2018; Kaji et al., 2018; Gill and Sillitoe, 2019; Morigaki et al., 2021; Gill et al., 2023). Our results showed that PACT/RAX was abundantly expressed in the cerebellum (Fig. 6A,B). Notably, we observed the highest concentration of PACT/RAX in the Purkinje neuron layer (Fig. 6A, brown staining; Fig. 6B, red fluorescence). Considering this observation, we further evaluated the expression of PACT/RAX in Purkinje neurons by performing double immunostaining for the Purkinje neuron-specific marker, calbindin (green), and PACT/RAX (red), with nuclear stain 4′,6-diamidino-2-phenylindole (DAPI; blue) (Fig. 6B). The colocalization of red and green fluorescence and presence of yellow fluorescence in the Purkinje neurons confirmed that PACT/RAX protein was expressed at high levels in these neurons.

### *Prkra^lear-5J^* mice show defects in cerebellar development and deficiencies in Purkinje neuron arborization

**Fig. 7.**
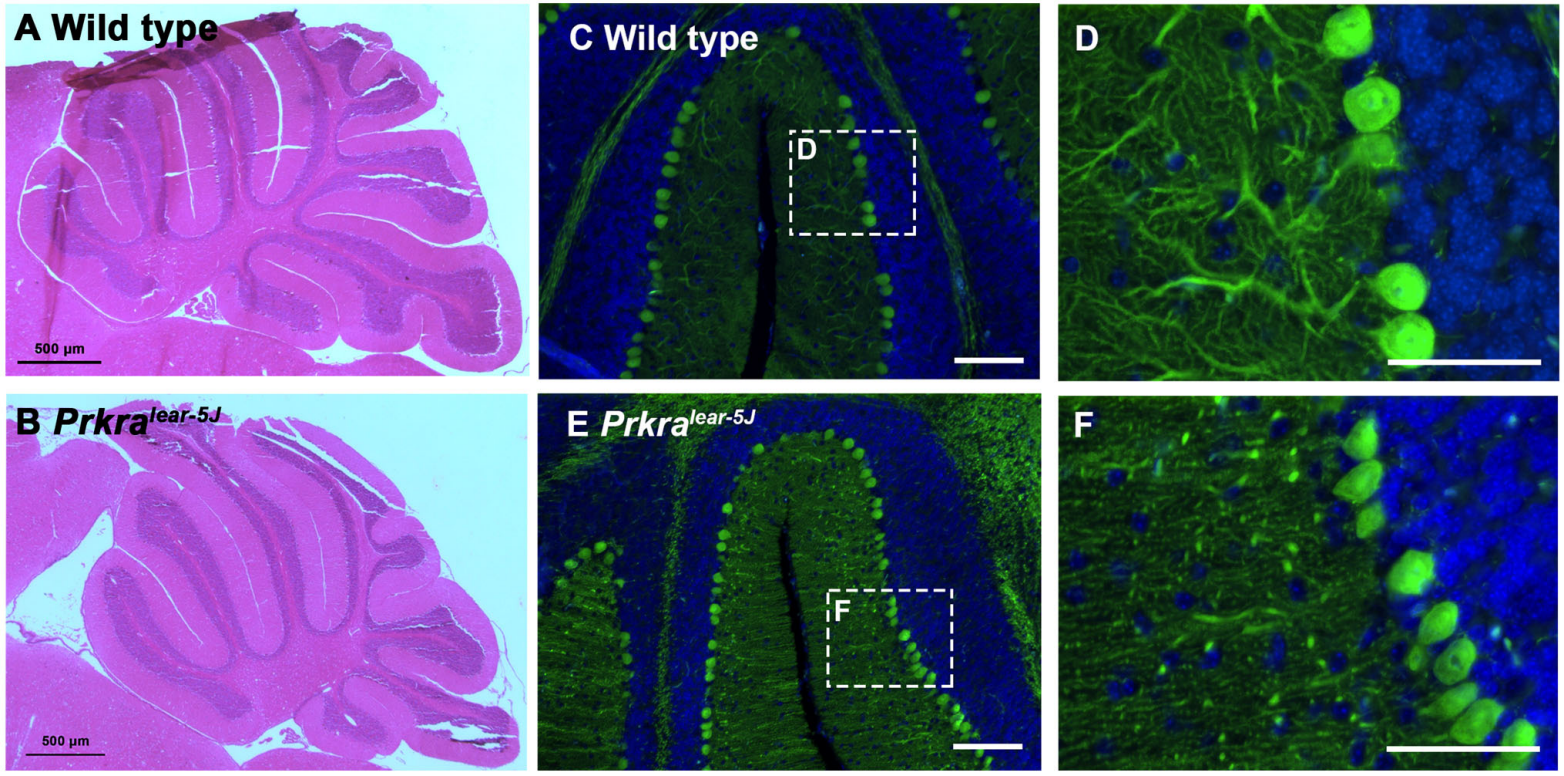
**The *Prkra^lear-5J^* mutation affects cerebellar development and reduces arborization in Purkinje neurons.** (A,B) Hematoxylin and Eosin staining on day 28 sagittal sections of wt (BTBR *T*^+^ *Itpr^tf^*/J) and *Prkra^lear-5J^* cerebellum. We examined six *Prkra^lear-5J^* mouse cerebellums, which exhibited varying degrees of reduced foliation. (C-F) Immunohistochemistry of day 28 sagittal sections of mouse cerebellar tissue stained for the Purkinje neuron marker, calbindin (green), and nuclear stain DAPI (blue). Dashed line boxes in C and E indicate areas magnified in D and F, respectively. Scale bars: 100 μm (C,E) and 20 μm (D,F).

The cerebellum of mammals is divided into ten distinct folia (Sillitoe and Joyner, 2007), After establishing that PACT/RAX is expressed in the mouse cerebellum, we assessed whether the *Prkra^lear-5J^* frameshift mutation has any effect on cerebellar folia. To address this, we compared mid-sagittal sections of wt (BTBR *T*^+^ *Itpr^tf^*/J) and *Prkra^lear-5J^* cerebellum derived from 28-day-old mice (two of each genotype) using Hematoxylin and Eosin (H&E) staining (Fig. 7A,B). In wt (BTBR *T*^+^ *Itpr^tf^*/J) and *Prkra^lear-5J^* mice, we observed the characteristic ten folia within the cerebellum, but the complexity of the folia was reduced in the *Prkra^lear-5J^* mice. The *Prkra^lear-5J^* folia demonstrated an elongated and less branched pattern relative to that of the BTBR *T*^+^ *Itpr^tf^*/J mice. The most distinguishing factor when comparing these foliations was within folium IX. In mice, this folium is subdivided into three lobules (IXa, IXb, IXc) (Sudarov and Joyner, 2007). Although we could observe these distinct lobules in the cerebellar tissue derived from wt mice, we did not detect such lobules in the *Prkra^lear-5J^* cerebellum. These initial observations indicated that the cerebellar development of *Prkra^lear-5J^* mice is affected, noting that the *Prkra^lear-5J^* brains that we evaluated were always smaller than those of the control mice, although there was some variation between samples. Owing to the abundance of PACT/RAX in the Purkinje cell layer seen in Fig. 6, we next determined whether there were any significant differences in Purkinje neurons between wt (BTBR *T*^+^ *Itpr^tf^*/J) and *Prkra^lear-5J^* mice. We performed immunostaining on sagittal sections of the cerebellar tissue using anti-calbindin antibody (green) to specifically mark Purkinje neurons and DAPI (blue) as the nuclear stain (Fig. 7C-F). The overall organization of Purkinje neurons was similar in both cerebellum sections (Fig. 7C,E). In wt (BTBR *T*^+^ *Itpr^tf^*/J) mice, Purkinje neurons demonstrated the characteristic well-branched arborization from the cell body (Fig. 7D). However, in the *Prkra^lear-5J^* cerebellum, we identified a dramatic reduction in the dendritic branching of Purkinje neurons (Fig. 7F) compared to that in the control cerebellum.

### *Prkra^lear-5J^* cerebellum shows a significant reduction in eIF2α phosphorylation

**Fig. 8.**
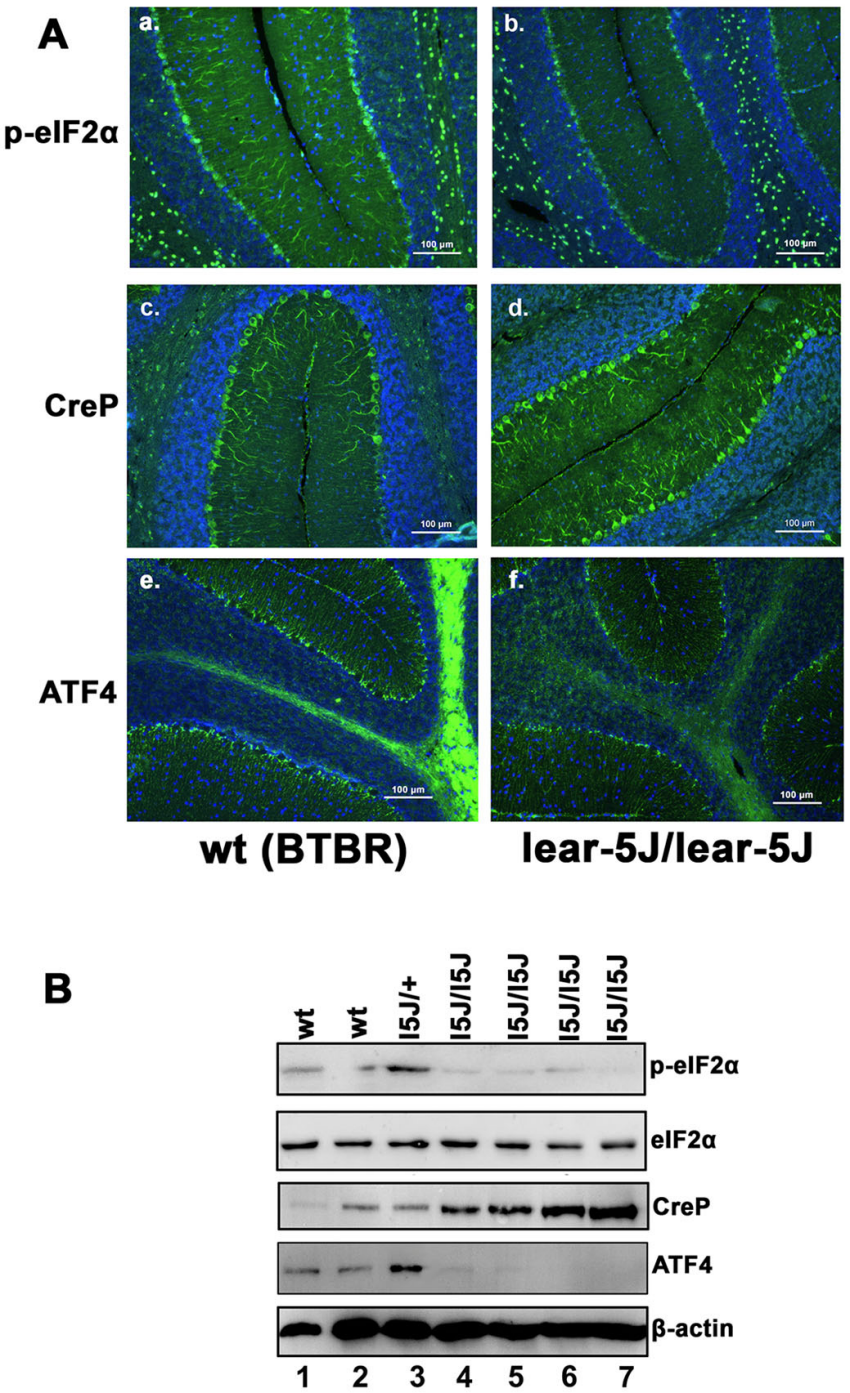
***Prkra^lear-5J^* cerebellum exhibits dysregulation of eIF2α phosphorylation.** (A) Immunohistochemistry of day 28 sagittal sections of wt (BTBR *T*^+^ *Itpr^tf^*/J) (a,c,e) and *Prkra^lear-5J^* (b,d,f) mouse cerebellum stained with the nuclear marker DAPI (blue) and for the protein of interest (green). a and b, DAPI (blue) and phosphorylated eIF2α (green); c and d: DAPI (blue) and CreP (green); e and f, DAPI (blue) and ATF4 (green). (B) Western blot analysis of wt (BTBR *T*^+^ *Itpr^tf^*/J) and *Prkra^lear-5J^* cerebellar extracts. Cerebellar extracts from two wt (BTBR *T*^+^ *Itpr^tf^*/J) (lanes 1 and 2), one *Prkra^lear-5J/+-^* heterozygous (lane 3) and four *Prkra^lear-5J/lear5-J^* homozygous (lanes 4-7) mice collected on day 28 were analyzed with antibodies against phospho-eIF2α (p-eIF2α), total eIF2α, CreP, ATF4 and β-actin. The best of three representative blots for each is shown.

A perturbation of the basal eIF2α phosphorylation levels, as well as dysregulation of stress-induced phosphorylation, has been previously linked to DYT-PRKRA (Vaughn et al., 2015; Burnett et al., 2019; Burnett et al., 2020) and also to other types of dystonia (Rittiner et al., 2016; Beauvais et al., 2018; Zakirova et al., 2018). As PACT/RAX-mediated regulation of PKR kinase activity impacts eIF2α phosphorylation, we next determined whether the *Prkra^lear-5J^* mutation resulted in any changes in eIF2α phosphorylation in the mouse cerebellum. To answer this question, we performed immunostaining on mid-sagittal sections of mouse cerebellum (Fig. 8A), probing with antibodies that specifically detect phosphorylated eIF2α, CreP (the constitutively expressed phosphatase that dephosphorylates eIF2α) and the ISR marker ATF4, which is synthesized in response to phosphorylated eIF2α. Our results indicated that mice homozygous for the *Prkra^lear-5J^* mutation showed a dramatic reduction in levels of phosphorylated eIF2α compared to those in wt (BTBR *T*^+^ *Itpr^tf^*/J) controls (Fig. 8Aa,b). Interestingly, our results also indicated that the *Prkra^lear-5J^* mice had elevated CreP levels compared to those of the wt controls (Fig. 8Ac,d), which might contribute to the presence of low levels of phosphorylated eIF2α in addition to inhibition of PKR by the lear-5J protein. Consequently, the levels of ATF4, which is a transcription factor and an effector of ISR that is synthesized only under conditions of eIF2α phosphorylation, were reduced in *Prkra^lear-5J^* mice compared to the wt controls (Fig. 8Ae,f). Furthermore, western blot analysis confirmed that cerebellar extracts from the *Prkra^lear-5J^* homozygous mice (Fig. 8B, lanes 4-7) had significantly less eIF2α phosphorylation than did the extracts from the wt mice (BTBR *T*^+^ *Itpr^tf^*/J; Fig. 8B, lanes 1 and 2). Additionally, the levels of CreP were higher in cerebellar extracts from *Prkra^lear-5J^* homozygous mice (lanes 4-7) than in those from wt mice (Fig. 8B, lanes 1 and 2). The levels of ATF4 followed the eIF2α phosphorylation levels and were significantly lower in *Prkra^lear-5J^* homozygous mice (Fig. 8B, lanes 4-7) than in the wt controls (Fig. 8B, lanes 1 and 2). These western blot results matched the immunostaining data in Fig. 8A and confirmed that the *Prkra^lear-5J^* homozygous mice had lower levels of eIF2α phosphorylation and ATF4, but higher levels of CreP phosphatase, in the cerebellum than did the wt controls. Interestingly, the *Prkra^lear-5J^* heterozygous cerebellar extract (Fig. 8B, lane 3) showed eIF2α phosphorylation levels higher than those of both the wt controls (Fig. 8B, lanes 1 and 2) and the *Prkra^lear-5J^* homozygotes (Fig. 8B, lanes 4-7). The reasons for this are currently unclear and will require additional analysis. Taken together with the results in Fig. 3 and Fig. 8A, these observations indicate that the lower levels of eIF2α phosphorylation in *Prkra^lear-5J^* cerebellum could result from inhibition of PKR by *Prkra^lear-5J^*-derived protein as well as higher expression of CreP.

## DISCUSSION

PACT/RAX serves as a negative regulator of general protein synthesis under conditions of cellular stress by triggering eIF2α phosphorylation via PKR activation (Ito et al., 1999; Patel et al., 2000; Bennett et al., 2004, 2006; Singh et al., 2009; Chukwurah and Patel, 2018). Our previous research has established that several DYT-PRKRA variants lead to enhanced PKR activation and eIF2α phosphorylation in response to ER stress. The recessively inherited P222L variant leads to delayed but heightened and persistent PKR activation and eIF2α phosphorylation to increase cell susceptibility to ER stress (Vaughn et al., 2015). A dominantly inherited frameshift variant causes insoluble protein aggregates, PKR activation and significant apoptosis even in the absence of stress (Burnett et al., 2019). Additionally, three other recessive and two dominant variants also lead to enhanced PKR interaction and activation, causing increased eIF2α phosphorylation and sensitivity to ER stress in DYT-PRKRA patient cells (Burnett et al., 2019). In addition to in our studies on DYT-PRKRA, maladaptive eIF2α signaling is seen in DYT-TOR1A (DYT1), DYT-THAP1 (DYT6) and sporadic cervical dystonia (Rittiner et al., 2016; Beauvais et al., 2018; Zakirova et al., 2018). Thus, dysregulated eIF2α phosphorylation has emerged as a convergent theme in dystonia.

In the present study, our objective was to determine whether the murine *Prkra^lear-5J^* mutation results in dysregulated eIF2α signaling, contributing to dystonia-like symptoms. A characterization of the biochemical properties of the truncated lear-5J protein revealed that it has reduced ability to bind dsRNA while retaining its ability to interact with PKR. This was expected, as we have previously characterized that the high-affinity binding to dsRNA requires two intact dsRBMs (Huang et al., 2002) and the lear-5J protein has lost the carboxy terminal part of dsRBM2 critical for binding to dsRNA. Based on our previous research, as the interaction of PACT/RAX with PKR is primarily mediated via the dsRBM1 (Chukwurah et al., 2018), the lear-5J protein retains its ability to interact with PKR. For PACT/RAX to activate PKR, the intact M3 motif is essential, and we and others previously characterized that deletion of M3 renders PACT/RAX unable to activate PKR despite its ability to interact with PKR (Peters et al., 2001; Huang et al., 2002). Thus, it is not surprising that lear-5J protein has lost its ability to activate PKR, although it can interact with PKR. Additionally, lear-5J protein inhibits PKR in the presence of both activators, dsRNA or PACT/RAX, which is likely due to direct binding of lear-5J protein to PKR, thus preventing its interaction with dsRNA or PACT/RAX. Alternatively, lear-5J protein could also directly bind to dsRNA and wt PACT/RAX protein to prevent their interaction with PKR, resulting in the observed inhibition of PKR kinase activity. The truncated lear-5J protein is not expected to activate PKR under conditions of stress in the brain. Phosphorylation of PACT/RAX on serine 287 after ER stress is essential for PKR activation (Peters et al., 2006; Singh et al., 2011; Singh and Patel, 2012), and, as serine 287 is lacking in lear-5J protein, lear-5J protein is expected to be unable to activate PKR after ER stress.

The presence of lear-5J protein in the brain, but not in MEFs, indicates that the truncated mutant protein may be degraded efficiently in MEFs but not in the brain. The lear-5J mRNA could be detected in brain and in MEFs, indicating that it is not degraded efficiently by NMD in both tissues. The efficiency of NMD is dependent on many factors depending on the location of the nonsense mutation, cell type and the mutated gene (Miller and Pearce, 2014). Thus, it is not surprising to detect the mRNA encoding lear-5J protein at variable levels in the brain and MEFs. It is worth noting that we have previously characterized the PACT/RAX-null MEFs to be significantly resistant to apoptosis in response to the ER stressor tunicamycin (Singh et al., 2009). Resistance of *Prkra^lear-5J^* MEFs to ER stress observed in the current study thus agrees with previous studies that the absence of PACT/RAX confers resistance to ER stress-induced apoptosis.

Truncated lear-5J protein being present in the brain may also be one possible reason for the dystonia phenotype observed in the *Prkra^lear-5J^* homozygous mice but not in *Prkra*^−/−^ mice. Rowe et al. previously described a *Prkra*^−/−^ mouse generated by targeting a 3′ exon that does not demonstrate any dystonia-like symptoms, but shares many overlapping developmental phenotypes with the *Prkra^lear-5J^* mice (Rowe et al., 2006; Dickerman et al., 2015; Palmer et al., 2016). Both *Prkra*^−/−^ and *Prkra^lear-5J^* mice exhibit reduced body size, craniofacial abnormalities, underdeveloped or small external ears, and hearing loss (Rowe et al., 2006; Dickerman et al., 2015). Another contributing factor to the lack of a dystonia phenotype in *Prkra*^−/−^ mice could be that it was produced in a different genetic background. The *Prkra*^−/−^ mice are described on C57BL/6 background, whereas the *Prkra^lear-5J^* mice are on the BTBR *T*^+^ *Itpr3^tf^*/J background (Rowe et al., 2006; Palmer et al., 2016). The BTBR *T*^+^ *Itpr3^tf^*/J genetic background contains a mutation in the *Itpr3* gene, which codes for inositol 1,4,5-triphosphate receptor type 3. This receptor plays a critical role in regulating intracellular calcium levels (Mangla et al., 2020), and, as calcium signaling is directly linked to ER homeostasis and eIF2α signaling, it is reasonable to speculate that the combination of calcium signaling dysregulation and *Prkra^lear-5J^* mutation may result in the enhanced dystonia phenotype, especially because we have previously linked PACT/RAX-mediated PKR regulation with a central role in cellular fate in response to ER stress (Singh et al., 2009; Vaughn et al., 2015; Burnett et al., 2019). A previous study demonstrated that the BTBR mice exhibit abnormal cerebellar development, leading to motor dysfunction (Xiao et al., 2020). This study noted increased proliferation of cerebellar granule neurons with enhanced cerebellar foliation, and hypertrophy of Purkinje cells with increased dendritic spines, in BTBR mice. The cerebellar defects observed in *Prkra^lear-5J^* mice differ markedly from those observed in the BTBR strain, suggesting that the truncated PACT/RAX protein has a specific effect. One limitation of our current study is the small sample size for our cerebellar morphology and immunohistochemical analyses owing to the limited availability of *Prkra^lear-5J^* homozygous mice (Palmer et al., 2016). The cerebellar morphology phenotype was variable among the four *Prkra^lear-5J^* homozygotes that we examined but was consistently different from that of wt. Thus, the observed reduced overall cerebellar volume, folia branching and dendritic branching should be regarded as descriptive study results, which in future can be followed by in-depth analysis of additional samples. The *Prkra^lear-5J^* homozygous mice develop dystonia at ∼2 weeks of age without any discernible external stressors, which indicates that changes in eIF2α phosphorylation in the brain triggered by the truncated lear-5J protein may directly lead to dystonia symptoms. Rapid changes in eIF2α phosphorylation are used for normal neuronal responses rather than strictly for the canonical stress response in other cell types (Bellato and Hajj, 2016), which may explain the dystonia onset in the *Prkra^lear-5J^* homozygous mice.

Lack of PACT/RAX function was also investigated by Bennett et al. (2008) in flies and mice. Additionally, a mouse missense mutation S130P in the second dsRBM of PACT/RAX resulted in defects in ear development, growth, craniofacial development and ovarian structure, but no movement disorder phenotype in mice with a C57BL/6J and C3HeB/FeJ mixed background (Dickerman et al., 2011). These results indicate that the genetic background can significantly alter the neuromuscular effects of PACT/RAX mutations. In future studies, it will be beneficial to breed the *Prkra^lear-5J^* mutation, as well as other DYT-PRKRA mutations, in a C57BL/6 genetic background, which could clarify whether the resulting dystonia phenotype arises owing to the truncated PACT/RAX protein alone or a combination of *Prkra^lear-5J^* mutation and the BTBR *T*^+^ *Itpr3^tf^*/J genetic background. As PACT/RAX is an evolutionarily conserved protein, its function has been studied in *Drosophila*. Flies carrying an inactivating transposon insertion in *Drosophila PACT/RAX* (also known as *loqs*/R3D1) caused highly abnormal commissural axon structure of the central nervous system and severe defect in neuromuscular coordination (Bennett et al., 2008), thus indicating that PACT/RAX may regulate motor coordination via mechanisms other than regulation of PKR as there is no PKR homolog in flies.

The cerebellum is a region of the brain that is indispensable for motor control (Timmann et al., 2010; Welniarz et al., 2021). When comparing the overall cerebellar developmental pattern of the *Prkra^lear-5J^* mice to that of wt (BTBR *T*^+^ *Itpr3^tf^*/J) mice, an overall reduction in the complexity of the foliation and a severe deficiency in the arborization of the Purkinje neurons is noted. Purkinje neurons facilitate communication between various cerebellar regions to control the final output in regulation of dexterous limb movements through their extensive dendritic arborization (Thanawalla et al., 2020; Gaffield et al., 2022). Interestingly, previous studies have specifically identified that the dysregulation of either sodium or potassium pumps in Purkinje neurons leads to the rapid onset of dystonia and parkinsonism or ataxia (Calderon et al., 2011; Tara et al., 2018). Our results suggest that the Purkinje neurons in *Prkra^lear-5J^* mice may have compromised functionality because of the severe deficit of dendritic arborizations, and that could contribute to the underlying dystonia phenotypes. Interestingly, perturbations in eIF2α phosphorylation have been linked to dysregulation of dendritic arborization in flies (Tsuyama et al., 2017). Previously, it has also been reported that PACT/RAX is essential for the migration of cerebellar granule neurons in the developing mouse cerebellum (Yong et al., 2015), thus indicating that PACT/RAX may regulate cerebellar development at multiple levels. Although cerebellar dysfunction has been increasingly described in dystonia, it could be worthwhile to compare the basal ganglia of *Prkra^lear-5J^* and wt mice in the future for any functional and/or structural alterations, as abnormal functioning of basal ganglia leads to loss of coordinated movements (Thomsen et al., 2024).

As perturbation of eIF2α signaling has emerged as a convergent mechanism for various forms of monogenic dystonia (Gonzalez-Latapi et al., 2021), our study highlights the importance of characterizing the mechanistic details underlying the resulting changes in eIF2α phosphorylation status and ultimately the regulation of ISR. More specifically, in the case of *Prkra^lear-5J^* mice, there is a reduction in the basal eIF2α phosphorylation levels, but the CreP phosphatase levels are elevated (Hicks et al., 2023). The inhibition of PKR activity by lear-5J truncated mutant protein and increased CreP levels could both contribute to the observed reduction in basal eIF2α phosphorylation. This contrasts with the increased phosphorylation of eIF2α phosphorylation, heightened PKR kinase activity and enhanced sensitivity to ER stress in DYT-PRKRA patient cells (Vaughn et al., 2015; Burnett et al., 2020). Additionally, similar increases in PKR activity and eIF2α phosphorylation was reported in DYT-EIF2AK2 (DYT33) patients carrying PKR missense variants with early-onset generalized dystonia (Kuipers et al., 2021). In support of our current findings, which are in contrast to those of our own previous studies with DYT-PRKRA patient cells, there are studies that describe reduced eIF2α phosphorylation as being detrimental to coordinated muscle movement. The most compelling and recent evidence comes from studies on DYT-TOR1A (DYT1), where a genome-wide RNA interference screen indicated a possible pathogenic role of deficient eIF2α signaling (Rittiner et al., 2016). Patient-derived cells and a mouse model supported both a pathogenic role and therapeutic potential for eIF2α pathway perturbations for DYT-TOR1A. Additionally, genetic and functional evidence is reported of a similar eIF2α pathway impairment in patients with sporadic cervical dystonia, owing to a rare variant in ATF4. The therapeutic potential of compounds that boosted the ISR showed corrective activity in cell and animal models of DYT-TOR1A (Caffall et al., 2021), further indicating that therapeutic manipulation of the eIF2α pathway could be beneficial for dystonia patients. In this study, the human immunodeficiency virus (HIV) protease inhibitor, ritonavir, corrected the variant TOR1A protein mislocalization *in vitro* and, when administered during an early postnatal period, showed therapeutic effects in a mouse model of DYT-TOR1A, reducing brain irregularities and ameliorating the dystonia phenotype. However, such manipulations must be controlled carefully in patients because both high and low eIF2α phosphorylation levels have been reported in various types of monogenic dystonia. In DYT-PRKRA, we have seen higher levels of eIF2α phosphorylation in patient-derived cells (Vaughn et al., 2015; Burnett et al., 2020), while lower eIF2α phosphorylation is seen in *Prkra^lear-5J^* mice that develop dystonia. Thus, boosting eIF2α phosphorylation could work in alleviating dystonia symptoms in cases in which ISR is attenuated such as DYT-TOR1A and sporadic cervical dystonia (Rittiner et al., 2016). It is also important to note that in the case of DYT-EIF2AK2 (DYT33), PKR-inactivating variants were reported in some patients (Mao et al., 2020), thereby suggesting that a reduction in PKR activity and consequently reduced eIF2α phosphorylation could also lead to dystonia pathophysiology. Furthermore, selective deletion of PERK, one of the eIF2α kinases, in mouse midbrain dopaminergic neurons resulted in multiple cognitive and age-dependent motor phenotypes, which could also be observed by expression of a phospho-mutant eIF2α (Longo et al., 2021). It is likely that precise regulation of the extent and duration of eIF2α phosphorylation is essential for optimal neuronal regulation of motor control, and either reduction or elevation of the ISR response may lead to lack of motor coordination. Thus, any future treatment options that target eIF2α phosphorylation would need to be developed with caution, keeping in mind that both too little and too high eIF2α phosphorylation can be detrimental to coordinated muscle movement. Recently, it was reported that the cholinergic neurons constitutively engage the ISR for dopamine modulation and skill learning (Helseth et al., 2021). Although phosphorylation of eIF2α has classically been viewed as a stress response, eIF2α phosphorylation-mediated regulation of protein synthesis is utilized by neurons for mechanisms besides stress response that include behavior, memory consolidation, neuronal development and motor control (Bellato and Hajj, 2016). Future research using targeted variants in specific neuronal subtypes can test the exact contribution of ISR and, specifically, eIF2α phosphorylation to neuronal control of muscle movement.

In conclusion, we identify that reduced eIF2α phosphorylation resulting from a frameshift mutation in murine *Prkra* gene affects cerebellar development. This study, combined with our earlier studies with DYT-PRKRA patient cells, provides direct evidence for a key role of a dysfunctional eIF2α pathway in the pathogenesis of dystonia. The *Prkra^lear-5J^* mice can also be a suitable mouse model for screening potential therapeutic options to alleviate the dystonia symptoms arising from dysregulated eIF2α signaling and should be tested for suitability for such studies.

## MATERIALS AND METHODS

### Cell lines and antibodies

HeLa M (American Type Culture Collection, CCL-2) and MEF (Murray Laboratory, The Jackson Laboratory) cell lines were cultured in Dulbecco's modified Eagle medium containing 10% fetal bovine serum and penicillin/streptomycin. All transfections were carried out using Effectene transfection reagent (Qiagen) as per the manufacturer’s protocol. Antibodies used were as follows: anti-PKR (human) monoclonal (R&D Systems, 71/10), anti-phospho-eIF2α (Ser-51) polyclonal (Cell Signaling Technology, 9721), anti-PACT polyclonal (ProteinTech, 10771-1-AP), anti-ATF4 monoclonal (Cell Signaling Technology, 11815), anti-Flag monoclonal M2-HRP (Sigma-Aldrich, A8592), anti-β-actin-peroxidase monoclonal (Sigma-Aldrich, A3854) and PPP1R15B rabbit polyclonal (ProteinTech, 14634-1-AP) (for CreP).

### *Prkra^lear5-J^* mice and tissues

Generation and maintenance of *Prkra^lear5-J^* mice was as described previously (Palmer et al., 2016). Mice were housed in 51 inch^2^ polycarbonate boxes on sterilized Northern White Pine bedding under 14 h:10 h light:dark cycles. Autoclaved NIH 31 (6% fat diet, Ca:P of 1.15:0.85, 19% protein, vitamin and mineral fortified; Purina Mills International, Richmond, IN, USA) grain and water acidified with HCl to achieve a pH of 2.8-3.2 were freely available. Mouse colony maintenance and use were reviewed and approved by The Jackson Laboratory Institutional Animal Care and Use Committee and in accordance with the National Institutes of Health guidelines for the care and use of animals in research. Brain tissues were collected from *Prkra^lear5-J^* and BTBR *T*^+^ *Itpr3^tf^*/J mice. The MEFs were generated from embryonic day (E)14.5 embryos from *Prkra^lear5-J^* intercrosses.

### Generation of lear-5J frameshift mutation

We generated the lear-5J frameshift mutation using site-specific mutagenesis through PCR amplification such that antisense primer contained a single adenine insertion relative to the *Prkra* template DNA. The following site-specific mutagenic primers were used: lear-5J sense, 5′-GCCTCGAGCACATATGTCCCATAGCAGGCATCG-3′; lear-5J antisense, 5′-GCGGATCCGAAATATTACTAAACTTGGCGAGAAATTTCTCAGCAGCATTTCTCTTGGCTTGTTTTTTTTGATGCCCCCTTTC-3′.

The subsequent PCR product was then ligated into pGEMT-Easy vector (Promega), and the presence of the mutation was validated through DNA-sequencing services provided by Eton Biosciences. After sequence validation, we generated amino terminal Flag-tagged lear-5J constructs in pBSIIKS+ using NdeI and BamHI restriction sites. We next subcloned the Flag-lear-5J ORF into the mammalian expression construct, pCDNA3.1, using XbaI-BamHI restriction sites.

### Co-IP assays

HeLa M cells were seeded at 20% confluency in six-well dishes 24 h prior to transfecting 500 ng of either Flag-wt PACT or Flag-lear-5J constructs using Effectene transfection reagent (Qiagen). Cells were harvested 24 h post-transfection and harvested in IP buffer (20 mM Tris-HCl pH 7.5, 150 mM NaCl, 1 mM EDTA, 1% Triton X-100, 20% glycerol). Whole-cell extract was then immunoprecipitated overnight at 4°C on a rotating wheel using anti-PKR antibody (R&D Systems, 71/10; 1:2000) conjugated to protein A sepharose beads (GE Healthcare) in IP buffer. Immunoprecipitates were then washed three times in IP buffer followed by resuspension and boiling for 5 min in 2× Laemmli buffer (150 mM Tris-HCl pH 6.8, 5% SDS, 5% β-mercaptoethanol, 20% glycerol). Samples were then resolved on 12% SDS-PAGE denaturing gels and transferred onto PVDF membranes. Blots were initially probed for Flag-PACT/lear-5J constructs (co-IP) using anti-Flag antibody (described above; 1:2000) followed by incubation in anti-PKR antibody (described above; 1:2000) to determine whether equal amounts of PKR were immunoprecipitated. Input blots of whole-cell extract without immunoprecipitation are shown to indicate equal amounts of protein in each sample.

### dsRNA-binding assay

wt PACT and lear-5J constructs in pCDNA3.1 were *in vitro* translated using the TNT-T7-coupled rabbit reticulocyte system from Promega while incorporating an ^35^S-methionine radiolabel, and dsRNA-binding ability was measured using poly(I:C)-conjugated agarose beads (Sigma-Aldrich). We diluted 4 μl of the *in vitro* translation product in 25 μl binding buffer (20 mM Tris-HCl pH 7.5, 0.3 M NaCl, 5 mM MgCl_2_, 1 mM DTT, 0.1 mM PMSF, 0.5% NP-40, 10% glycerol), incubated in 25 μl poly(I:C)-agarose beads and incubated at 30°C for 30 min. We then washed the beads four times with 500 μl binding buffer, and bound proteins were analyzed via SDS-PAGE gel electrophoresis and autoradiography. The competition assay was performed incubating either soluble ssRNA, poly(C), or dsRNA, poly(I:C), with the poly(I:C)-agarose beads before the adding the *in vitro* translated proteins. To ensure that the presence of PACT was due to the dsRNA-binding capacity, we assayed *in vitro* translated ^35^S-methionine-labeled firefly luciferase, which has no dsRNA-binding ability. Bands in bound and total lanes were quantified using a Typhoon FLA7000 PhosphorImager (GE Healthcare) by analyzing the relative band intensities of the total and bound lanes. Percentage of PACT bound to beads was calculated and plotted.

### Expression and purification of PACT/lear-5J from *Escherichia coli*

The ORFs of wt PACT and lear-5J frameshift mutation were subcloned into pET15b (Novagen) to generate an in-frame fusion protein with a histidine tag. Recombinant proteins were then expressed and purified as previously described (Patel and Sen, 1998).

### PKR activity assay

HeLa M cells were treated with IFNβ for 24 h and harvested at 70% confluency. The cells were washed using ice-cold PBS and centrifuged at 600 ***g*** for 5 min. They were then resuspended in lysis buffer (20 mM Tris-HCl pH 7.5, 5 mM MgCl_2_, 50 mM KCl, 400 mM NaCl, 2 mM DTT, 1% Triton X-100, 100 U/ml aprotinin, 0.2 mM PMSF, 20% glycerol) and incubated on ice for 5 min. Whole-cell lysates were centrifuged at 10,000 ***g*** for an additional 5 min. PKR was then immunoprecipitated from 100 μg of this lysate using anti-PKR monoclonal antibody (R&D Systems, MAB1980; 1:2000) in a high-salt buffer (20 mM Tris-HCl pH 7.5, 50 mM KCl, 400 mM NaCl, 1 mM EDTA, 1 mM DTT, 100 U/ml aprotinin, 0.2 mM PMSF, 20% glycerol, 1% Triton X-100) at 4°C on a rotating wheel for 30 min. We then added 10 µl protein A-sepharose beads to each immunoprecipitate followed by an additional 1 h incubation under the same conditions. The protein A-sepharose beads were then washed four times in high-salt buffer followed by an additional two washes in activity buffer (20 mM Tris-HCl pH 7.5, 50 mM KCl, 2 mM MgCl_2_, 2 mM MnCl2, 100 U/ml aprotinin, 0.1 mM PMSF, 5% glycerol). PKR activity assay using the PKR bound to protein A-sepharose beads was conducted by incorporating 500 ng purified eIF2α as the PKR substrate, 0.1 mM ATP, 10 μCi [γ-^32^P] ATP, and increasing amounts of either recombinant wt PACT or recombinant lear-5J (400 pg to 4 ng) as the PKR activator. The reaction mixture was then incubated at 30°C for 10 min, resolved on a 12% SDS-PAGE gel and analyzed via autoradiography.

### DNA fragmentation analysis

DNA fragmentation analysis was performed as described previously (Vaughn et al., 2015). MEFs (5×10^6^) established from wt and *Prkra^lear-5J^* mice were treated with 0.5 μg/ml tunicamycin for 48 h followed by DNA fragmentation analysis. To quantify the DNA fragmentation, the fluorescence image was inverted, and the total band intensities in the entire lanes were computed with ImageQuant software on a Typhoon FLA7000 PhosphorImager (GE Healthcare) and compared with untreated samples as well as between wt and patient cells. The band intensities in wt untreated samples were considered as 1.0, and fold increases in band intensities with respect to wt untreated samples were calculated and subjected to statistical analysis. A statistical analysis from four different experiments was performed to calculate *P*-values to determine significant differences between wt and untreated and treated patient samples.

### Caspase 3/7 activity assays

wt and *Prkra^lear-5J^* MEFs were seeded at a concentration of 300,000 cells/ml medium and treated with 5 μg/ml tunicamycin over a 36 h time course. Samples were collected at indicated time points and mixed with equal parts Caspase-Glo 3/7 reagent (Promega, G8090) and incubated for 45 min. Luciferase activity was measured and compared to cell culture medium alone and untreated cells as the negative controls. A statistical analysis from six different experiments was performed to calculate *P*-values to determine significant differences between wt and *Prkra^lear-5J^* samples.

### Western blot analysis

Protein extracts were prepared from a fraction of the brains of wt mice, mice heterozygous for the *Prkra^lear-5J^* mutation and mice homozygous for the *Prkra^lear-5J^* mutation using western lysis buffer (20 mM Tris-HCl pH 7.5, 5 mM MgCl_2_, 50 mM KCl, 400 mM NaCl, 2 mM DTT, 1% Triton X-100, 100 U/ml aprotinin, 0.2 mM PMSF, 20% glycerol) containing a 1:100 dilution of protease inhibitor (Sigma-Aldrich) and phosphatase inhibitor (Sigma-Aldrich). Tissue was initially homogenized in the lysis buffer cocktail described above and incubated on ice for 5 min followed by centrifugation at 13,000 ***g*** for 10 min. Concentration of total protein extract was then determined through Bradford assays, and appropriate amounts were resolved on SDS-PAGE denaturing gels to detect the proteins of interest. Protein extract derived from MEFs were prepared from wt and *Prkra^lear-5J^* MEFs cultured as described above. Cells were initially seeded at 40% confluency and harvested 24 h later using the lysis buffer cocktail described above. Concentrations of whole-cell extracts were determined by Bradford assays. Proteins were detected via chemiluminescence and the antibodies indicated above (1:500).

### RT-PCR

RNA was isolated from either (1) a fraction of mouse brain derived from wt mice, *Prkra^lear-5J^* heterozygous mice and *Prkra^lear-5J^* homozygous mice, or from (2) wt and lear-5J homozygous MEFs. In all cases, tissue or cells were incubated on ice for 5 min in RNA-Bee (Tel-Test, Inc.) and in the presence of chloroform:isoamylacetate (24:1). Lysates were then centrifuged at 12,000 ***g*** for 15 min at 4°C. The fraction of lysate containing RNA was then carefully collected and precipitated overnight in an equal volume of isopropanol at −20°C. Samples were centrifuged at 12,000 ***g*** for 15 min to pellet the RNA. Supernatant was removed, and the RNA pellet was washed 2× in 70% ETOH and centrifuged at 12,000 ***g*** for 8 min at 4°C followed by a 1 h incubation at room temperature to ensure that RNA was devoid of alcohol contamination. Purified RNA pellets were then resuspended in nuclease-free water. The cDNA was generated using 1 µg total RNA, 75 nG random hexamer and reverse transcriptase (Thermo Fisher Scientific) as per the manufacturer’s protocol. Finally, relative levels of *Prkra* mRNA were compared using *Prkra*-specific primers, and ribosomal protein S15 primers were used to amplify this housekeeping gene to ensure that equivalent amounts of cDNA were used in each reaction. The following primers were used: *Prkra* sense, 5′-ATGTCCCATAGCAGGCATCGTGCCG-3′; *Prkra* antisense, 5′-CCTTCCTGGGAAAGGGTATATTCAGG-3′; S15 sense, 5′-TTCCGCAAGTTCACCTACC-3′; S15 antisense, 5′-CGGGCCGGCCATGCTTTACG-3′.

### Histology and immunostaining

Cerebellums derived from day 28 *Prkra^lear-5J^* homozygous and wt (BTBR *T*^+^ *Itpr^tf^*/J) mice were fixed at 4°C overnight in 4% paraformaldehyde in PBS. The fixative was then removed, and the cerebellums were washed in PBS followed by dehydration in absolute ethanol. Following the dehydration step, tissues were permeabilized with methyl salicylate and embedded in paraffin. We generated 6 μm sagittal sections from each tissue and evaluated them through H&E staining. Immunostaining was carried out as previously described (Youngblood et al., 2018), and antibodies, dilutions and detection methods are indicated in Table S1. In brief, experiments utilizing mouse primary antibodies utilized blocking reagents from the M.O.M. kit (Vector Laboratories), and experiments utilizing primary antibodies from other species used the blocking reagent from Tyramide Signal Amplification (TSA) kit no. 22 dissolved in PBS (Thermo Fisher Scientific), as per the manufacturers’ protocols. In each case, primary antibodies were incubated overnight at 4°C in the appropriate blocking reagent. Samples were then incubated in species-specific secondary antibodies in TSA block for 30 min at room temperature. Biotin-conjugated secondary antibodies (Thermo Fisher Scientific) were incubated in streptavidin conjugates (Alexa Fluor 488, Alexa Fluor 594 or HRP; Thermo Fisher Scientific) at a 1:100 dilution in TSA block (Thermo Fisher Scientific) at room temperature for 30 min. Following this incubation, the streptavidin-HRP conjugates were either subjected to SigmaFast 3,3′-diaminobenzidine (DAB) staining (Sigma-Aldrich) or TSA with Alexa Fluor 488 (TSA kit no. 22, Thermo Fisher Scientific) for 3 min in each case. The sections were counterstained with 300 nM DAPI for 5 min prior to mounting in fluorescence mounting medium. DAB detection was counterstained with 0.5% Methyl Green in 0.1 M sodium acetate buffer (pH 4.2) for 5 min at room temperature.

## Supplementary Material

10.1242/dmm.050929_sup1 Supplementary information
