# Supplementary material for: Mutation in Prkra results in cerebellar abnormality and reduced eIF2α phosphorylation in a model of DYT-PRKRA
Source: Dis Model Mech. 2024 Nov 26;17(11):dmm050929. doi: 10.1242/dmm.050929 (PMC11625895; doi:10.1242/dmm.050929)
Supplement: Supplementary information [file dmm-17-050929-s1.pdf]

Table S1. Primary antibodies and detection methodology

Protein	Source	Dilution	Detection method
Calbindin	Abcam ab82812	1:300	Invitrogen goat anti-mouse secondary antibody, Alexa Fluor 488 or goat anti-rabbit secondary antibody, Alexa Fluor 594 for double immunostaining
PACT/RAX	ProteinTech 10771-1-AP	1:100	Invitrogen goat anti-rabbit secondary antibody, biotin followed by Invitrogen streptavidin, HRP, and DAB
p-eIF2α	CST #9721	1:100	Invitrogen goat anti-rabbit secondary antibody, biotin followed by Invitrogen streptavidin, Alexa Fluor 488
CreP	Abcam ab220949	1:200	Invitrogen goat anti-rabbit secondary antibody, biotin followed by Invitrogen streptavidin, Alexa Fluor 488
ATF4	CST #11815	1:500	Invitrogen goat anti-rabbit secondary antibody, biotin followed by Invitrogen streptavidin, Alexa Fluor 488
